# Prevalence of Major Risk Factors and Assessment of 10-Year Risk for Cardiovascular Diseases Among Adults in Yaoundé, Cameroon: A Cross-Sectional Study

**DOI:** 10.7759/cureus.71672

**Published:** 2024-10-17

**Authors:** Yves Wasnyo, Clarisse Mapa Tassou, Lambed Tatah, Camille Mba, Jean-Claude Mbanya, Eugene Sobngwi, Felix Assah

**Affiliations:** 1 Department of Public Health, Faculty of Medicine and Biomedical Sciences, University of Yaoundé I, Yaoundé, CMR; 2 Health of Population in Transition, University of Yaoundé I, Yaoundé, CMR; 3 Department of Public Health, Faculty of Medicine and Pharmaceutical Sciences, University of Dschang, Dschang, CMR; 4 Department of Public Health, Medical Research Council (MRC) Epidemiology Unit, University of Cambridge, Cambridge, GBR; 5 Department of Internal Medicine and Specialities, Faculty of Medicine and Biomedical Sciences, University of Yaoundé I, Yaoundé, CMR

**Keywords:** adult, cardiovascular disease risk, lifestyles, risk scores, yaounde

## Abstract

Objective

This study aimed to assess the prevalence of major risk factors for cardiovascular disease and the 10-year cardiovascular risk in an adult population residing in Yaoundé, Cameroon.

Methodology

We conducted a cross-sectional survey in 10 purposively selected neighbourhoods of Yaoundé, involving one adult per household who consented to participate. We collected data on personal and family history, lifestyle and nutritional habits, anthropometric parameters, and blood pressure, and calculated prevalence rates with 95% CI.

Results

The study involved 411 participants (172 males) with a median age of 28 years (IQR 23-40 years).

Prevalence of CVD risk factors in our study population were family history of heart attack (4.4%; 95% CI 2.8-6.8%), stroke (7.5%; 95% CI 5.3-10.5%), harmful alcohol consumption (40.4%; 95% CI 35.8-45.3%), current smoking (5.1%; 95% CI 3.3-7.7%), second-hand smoking (26.6%; 95% CI 22.6-31.1%), low physical activity (66.4; 95% CI 61.7- 70.8%), inadequate fruit/vegetable intake (36.6%; 95% CI 31.4-42.1%), self-reported anxiety (83.5%; 95% CI 79.6-86.8%) and depression (52.3%; 95% CI 47.4-57.1%), overweight (27.0%, 95% CI 22.9 to 31.5%), obesity (25.1%, 95% CI 21.1 to 29.5%), abdominal obesity (65.9%; 95% CI 61.1-70.2%), excess body fat (46.3%; 95% CI 41.4-51.1%), suspected prehypertension (58.3%; 95% CI 51.1-65%) and hypertension (41.8%; 95% CI 35-48.9%).

Furthermore, 40% of overall participants had a moderate-to-high 10-year CVD risk. Independent factors such as a personal history of other chronic diseases and parental history of either diabetes or hypertension combined with behaviours like harmful alcohol consumption and mean blood pressure might significantly influence the cardiovascular risk of our study population

Conclusion

In Yaoundé, Cameroon, many adults have major risk factors for cardiovascular disease, with nearly one-third of young adults at moderate to high risk of developing CVD within the next decade. Thus, it is crucial to implement targeted interventions to mitigate the risk of CVD.

## Introduction

Non-communicable diseases (NCDs) represent a significant global health burden, with a disproportionate impact on low-income populations. Poverty and inequality contribute to the rise of NCDs and exacerbate risk factors such as smoking, alcohol consumption, and poor diet, resulting in a higher disease burden among the urban poor [1]. This increase can be primarily attributed to a lack of awareness, inadequate treatment, and control measures for major CVD risk factors, including high salt consumption in food, insufficient physical activity levels, and harmful alcohol use [2]. Assessment and management methods for individuals at elevated risk of cardiovascular issues involve maintaining optimal blood pressure, monitoring blood sugar levels, and controlling BMI to prevent being obese or overweight [3]. Introducing intervention approaches from an early age can postpone the development of these conditions into adulthood [4].

It has been argued that focusing on individual risk factors can neglect societal and environmental influences on cardiovascular issues [5]. Rather than solely targeting blood pressure, blood sugar levels, and BMI, addressing economic inequality, access to healthy food options, and community-level interventions may have a more significant impact on preventing these health issues [6]. This approach would be particularly beneficial for developing countries, where rapid urbanisation is leading to unhealthy dietary and physical activity habits combined with environmental pollution that threatens cardiovascular health. Therefore, it is crucial to develop policies to efficiently tackle chronic disease morbidity, particularly in developing nations, where over 80% of cardiovascular-related deaths occur [7].

The accurate prediction of cardiovascular disease (CVD) risk is essential to initiate appropriate intervention, and the Non-Laboratory INTERHEART Modifiable Risk Score (NL-IHMRS) is a suitable tool to estimate future CVD risk. This tool was based on a multi-ethnic study sample from 52 countries worldwide [8]. It is superior to other models based on American (Framingham Risk Score) or mostly Caucasian populations (Systematic Coronary Risk Evaluation Score) having variable predictability in non-White populations [9]. The INTERHEART model works well in different ethnic and geographic groups and covers many factors [8]; therefore, it is more suitable and trustworthy in sub-Saharan African settings. Moreover, this tool is simple to use by non-medical personnel. The NL-IMHRS incorporates dietary patterns, physical activity, and psychosocial factors, which are the most significant behavioural risk factors for CVD

Among the numerous studies addressing cardiovascular disease risk in Cameroon, only one used the NL-IHMRS [10], indicating that it is still a relatively overlooked instrument. Furthermore, rather than the previous study using NL-IHMRS to assess global cardiovascular risk in a population of university students, this study will be the first to include an adult population with a wide age range. A comprehensive understanding of the prevalence of major cardiovascular risk factors, including harmful alcohol consumption, tobacco smoking, dietary habits, physical inactivity, mental health, metabolic syndrome, as well as the overall 10-year cardiovascular risk of an adult population in Yaoundé, is essential for the development of targeted prevention and control strategies. It would also provide valuable insights into the burden of cardiovascular diseases in this population.

## Materials and methods

Study design and setting

We conducted a community-based cross-sectional study among individuals sampled from 10 purposely selected neighbourhoods of Yaoundé, categorised by their level of deprivation, according to the Yaoundé City Council Director Urbanisation Plan, using the 2002 nomenclature that classified neighbourhoods as spontaneous, peri-urban, middle-standing, and high-standing, assuming that spontaneous is more deprived than middle- or high-standing neighbourhoods [11]. Table 1 presents key characteristics of the selected neighbourhoods.

**Table 1:** Distribution and deprivation level of selected neighbourhoods CCDUP: City Council Director Urbanisation Plan - Spontaneous habitation: Unplanned, informal settlements typically on urban outskirts, often with makeshift housing. - Middle standing: Housing for middle-income groups, offering moderate amenities and services. - High standing: Upscale, luxurious residential accommodations with premium facilities and services. - Periurban: Transitional zones between urban and rural areas, featuring mixed land use and diverse housing types.

Subdivision	Selected neighbourhoods	Typology (CCDUP 2002)	Deprivation
Yaoundé 1	Nkolondom	Spontaneous periurban	High
Yaoundé 2	Cite Verte	Middle standing	Low
Yaoundé 3	Obobogo	Spontaneous periurban	High
Yaoundé 4	Nkomo	Spontaneous periurban	High
	Ndamvout	High standing	Low
Yaoundé 5	Ngousso	Middle standing	Low
	Essos	Middle standing	Low
Yaoundé 6	Biyem Assi	Middle standing	Low
	Etoug Ebe	Spontaneous periurban	High
Yaoundé 7	Oyomabang	Spontaneous periurban	High

Sampling strategy

**Figure 1:**
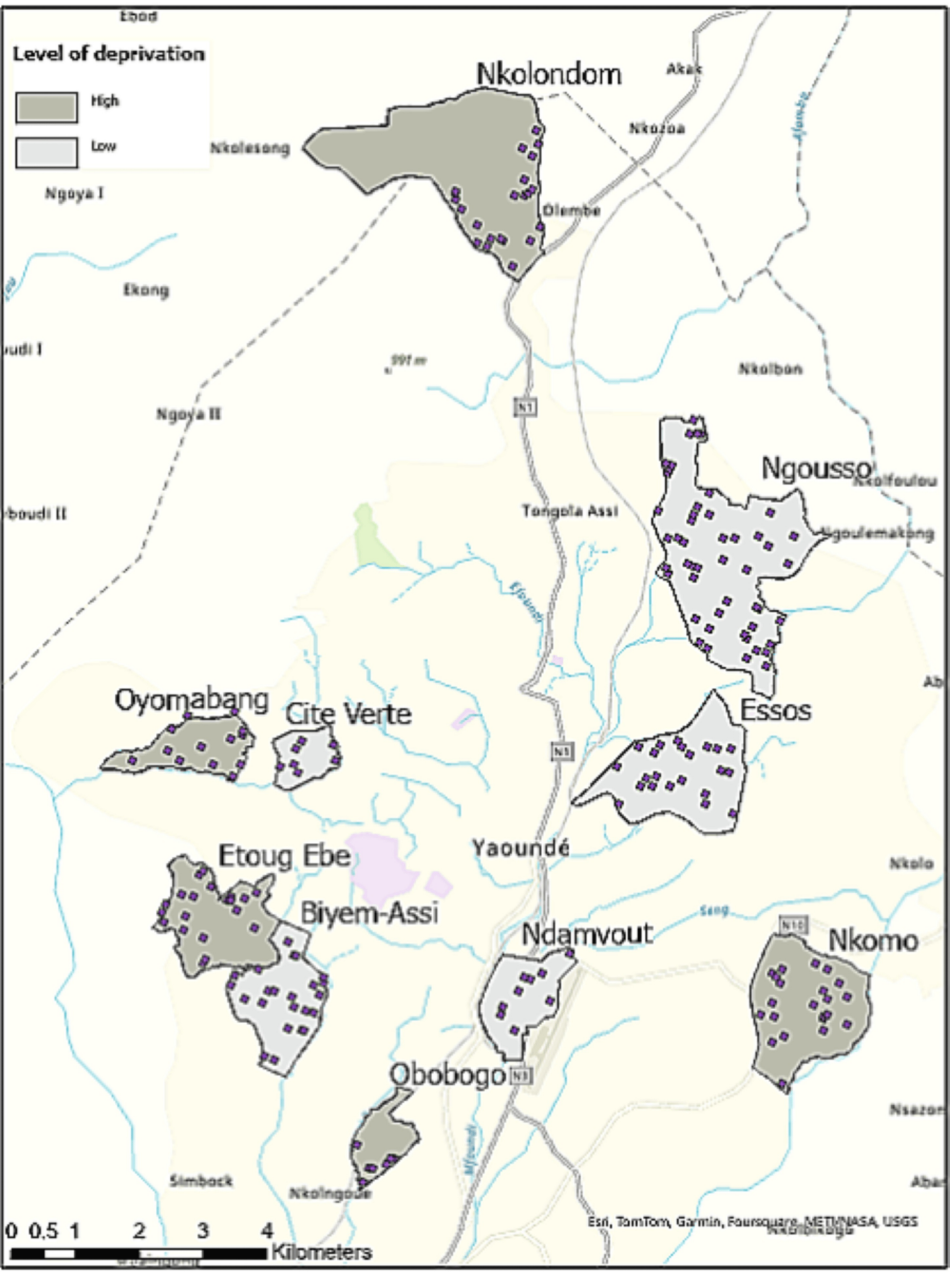
Map of the sampling points in selected neighbourhoods. This map was created by the authors for this study and indicates the neighbourhoods where individuals were sampled for the survey, as well as the relative locations and sizes of the areas of interest.

This study is nested in the GDAR Spaces study, which addresses the urbanisation and climate change syndemic and non-communicable disease risk [12]. According to the literature, the study audits 25% of existing routes in selected neighbourhoods [13], starting from a randomly selected household, sampled from Yaoundé’s buildings data [14]. These selected households constituted the sampling point of this study, around which two or three households were sampled (Figure 1). One adult (aged 18 years or older) per household was invited to participate.

Study participants

In each neighbourhood, we included one adult (18 years and older) per household, who had lived in the selected neighbourhoods for over a month and consented to participate. Participants who had any mobility impairment within the three months prior to the data collection were excluded from the study, as well as pregnant or breastfeeding participants.

We calculated the sample size (n) using the formula 

$n = \frac{p(1-p)}{i^2} \cdot Z^2_{(\alpha/2)}$

(p = the combined prevalence of both overweight and obesity prevalence as an indicator risk factor in the Yaoundé adult population, i = standard error of the proportion and = 1.96 the Z(α/2) value of t table for α=0.05). 

According to a systematic review of overweight and obesity epidemiology in Cameroon by Nansseu et al. in 2019, at least 26.0% (95%CI, 17.6-35.3%) of Cameroonian adults are overweight and 15.1% (95%CI 9.3-22.1%) are obese [15]. Accordingly, the minimum sample size was calculated to be 372 participants.

Data collection

We used a standardised, pretested questionnaire for data collection. It has three sections: sociodemographic characteristics, medical history (family and personal medical history, and lifestyle habits), and anthropometric and blood pressure (BP) measurements. After obtaining written consent and checking for eligibility, a questionnaire was administered.

Alcohol and Tobacco

We assessed alcohol consumption behaviour using the Alcohol Use Disorder Identification Test-Consumption (AUDIT-C) score; accordingly, we considered hazardous alcohol drinking based on an AUDIT-C score ≥4 for men and ≥3 for women [16].

The tobacco index was calculated by multiplying the number of packs smoked per day (20 cigarettes per pack) by the total number of years of smoking [17].

Fruit and Vegetable Consumption

The dietary recall method was used to collect information on the types, amounts, and servings of vegetables and fruits consumed in the last week. The measurement of the amount of fruit and vegetables was aided by pictorial cards and measuring cups (one standard serving of fruit or vegetables was equal to 80 g). We considered fruit intake adequate at 1.5-2 cups daily, while adequate vegetable intake was defined as 2-3 cups per day [18].

Physical Activity

We evaluated physical activity with the Global Physical Activity Questionnaire and categorized into four groups: "mainly sedentary" for those with no physical activity, even during leisure; "mild exercise" for easy walking of at least 10 minutes several times a week or moderate/strenuous exercise below WHO recommendations; "moderate exercise" for activities that slightly increase breathing or heart rate, done at least 30 minutes five days per week; and "strenuous exercise" referred to any activity that significantly raises breathing or heart rate, done for at least 25 minutes on a minimum of three days per week [19].

Anthropometric Measurements

Height was measured to the nearest 0.5 cm using a standard rigid stadiometer. In contrast, weight (to the nearest 0.1 kg) and fat mass percentage were measured with a body composition analyser (Tanita TBF-53 scales, Tanita UK, Yiewsley, Middlesex, UK). Body mass index (BMI) was determined by dividing weight (kg) by height squared (m²) and classified into four categories: underweight (<18.5), normal (18.5-24.9), overweight (25.0-29.9), and obese (≥30.0). Using a non-stretchable measuring tape, we measured mid-upper arm, waist, and hip circumferences to the nearest 0.1 cm. We measured the mid-upper arm circumference at the midpoint between the tips of the shoulder and elbow and the waist circumference (WC) at the midpoint between the top of the iliac crest and the lower margin of the last palpable rib in the mid-axillary line. The hip circumference was measured as the largest circumference of the buttocks. Therefore, we calculated the waist-to-hip ratio (WHR) as the waist circumference (cm) divided by the hip circumference (cm).

Excess body fat mass was defined at a fat mass over 20% for men and 33% for women, based on the body composition analyser cut-offs and abdominal obesity was defined according to the International Diabetes Federation (IDF) criteria (WC over or equal to 94 cm for men and 80 cm for women) [20], and according to the WHO criteria (WHR over or equal to 0.9 for men and 0.85 for women) [21].

Blood Pressure

Blood pressure (BP) was measured using an electronic sphygmomanometer (Omron M5-1, Omron Healthcare, Kyoto, Japan), before which each participant had at least a five-minute rest in a seated position, and the sphygmomanometer was calibrated. BP was measured twice on the left arm at five-minute intervals. The average of the two readings was used as the final measurement for the analysis. Suspected hypertension was identified by a systolic blood pressure (SBP) of 140 mmHg or higher, a diastolic blood pressure (DBP) of 90 mmHg or higher, or a self-reported history of antihypertensive medication use. Prehypertension was suspected when the participant had a SBP ranging from 120 to 139 mmHg or a DBP between 80 and 89 mmHg [22].

Calculation of NL-IHMRS

The global risk of CVD was determined using the Non-laboratory INTERHEART Modifiable Risk Score, a validated score for quantifying the risk factor burden and estimating the future risk of CVD without the use of laboratory testing. The non-laboratory-based tool has nine components: age and sex, smoking (active and passive), self-reported diabetes mellitus, self-reported hypertension, family history of cardiovascular disease (heart attack, parents), WHR, psychosocial factors (stress and depression), dietary factors, and leisure-time physical activity. The overall sum score of the NL-IHMRS ranges from 0 to 48, with higher scores reflecting a higher probability of developing CVD in the future. Based on previous studies, this score was further classified as low risk (score between 0 and 9), moderate risk (score between 10 and 15) and high risk (score between 16 and 48) [8]. The tool can be found in the supplementary table in Appendix 1 [9]. 

Statistical analysis

Data from anonymised questionnaires were coded, entered, cleaned, and double-checked using CsPro Version 7.0 (US Census Bureau, Washington, DC), and R version 4.4.1 (R Foundation for Statistical Computing, Vienna, Austria) served for data analysis. Summary statistics were presented as median (IQR) for continuous variables (due to skewed distributions) and frequency (percentage) for categorical variables. Variable comparisons were performed using the χ2 test or Fisher exact test, or non-parametric tests (Mann-Whitney U test) wherever applicable.

The strength and direction of any relationship between the quantitative variables were assessed using Spearman's correlation test for continuous variables and the Cramer V test for categorical variables. We considered that variables were highly correlated when the Spearman correlation coefficient (ρ) or Cramer correlation coefficient (V) were higher than +0.5 or lower than -0.5. The highly correlated variables were combined, categorised or dropped depending on their clinical relevance.

To assess the extent to which each of the various CVD risk factors influences the global cardiovascular risk, we proceeded with an ordinal logistic regression analysis. BMI, BP and significant variables from the univariable model were included in the multivariable model. The results were shown as odds ratio (OR) coefficients with their 95% confidence intervals (CI). None of the variables used to derive the NL-IHMRS were included in the regression model. Results were considered statistically significant at p < 0.05. 

## Results

General characteristics

A total of 411 individuals participated in this study, with a median age of 28 years (IQR, 23-40 years). Among the participants, 172 (41.6%) were male, 229 (55.7%) were aged 30 and under, almost all were Christians by faith (89.7%), and 51.8% were single. Most of the participants were from both Fang-Beti and Grassfield (84.5%) ethnic origins, and 18.5% had never attended school or attended primary school only. Table 2 shows the sociodemographic characteristics of the study population according to gender and age.

**Table 2:** Sociodemographic characteristics of the study population ^a^ For expected observations < 5 in any of the cells, chi-square estimates may be incorrect. IQR: interquartile range

Characteristics	Total	Gender			Age group		
		Male (N = 172)	Female (N = 239)	p-value	Up to 30 (N = 229)	Over 30 (N = 182)	p-value
Age (years), Median (IQR)	28 (23-40)	30.5 (23-42)	28 (23-37)	0.15	24 (21-27)	42 (35-54)	-
Age (years), n (%)				< 0.01	-		
Up to 30	229 (55.7)	86 (50.0)	143 (59.8)	-	-	-	-
31 to 40	83 (20.2)	31 (18.0)	52 (21.8)	-	-	-	-
Over 40	99 (24.1)	55 (32.0)	44 (18.4)	-	-	-	-
Level of study, n (%)				0.42			< 0.01
Never studied/primary	76 (18.5)	32 (18.6)	44 (18.4)	-	28 (12.2)	48 (26.4)	-
Secondary education	219 (53.3)	86 (50.0)	133 (55.7)	-	118 (51.5)	101 (55.5)	-
High school	116 (28.2)	54 (31.4)	62 (25.9)	-	83 (36.2)	33 (18.1)	-
Religion, n (%)				0.12^a^			0.33
Christian	369 (89.7)	151 (87.8)	218 (91.2)	-	202 (88.2)	167 (91.8)	-
Muslims	29 (7.1)	12 (7.0)	17 (7.1)	-	20 (8.7)	9 (5.0)	-
Other	13 (3.2)	9 (5.2)	4 (1.7)	-	7 (3.0)	6 (3.3)	-
Ethnic origin, n (%)				0.50^a^			< 0.01^a^
Sudano-Sahelian	43 (10.5)	18 (10.5)	25 (10,5)	-	36 (15.7)	7 (3.9)	-
Grassfield	147 (35.8)	69 (40.1)	78 (32.6)	-	82 (35.8)	65 (35.7)	-
Sawa	13 (3.2)	6 (3.5)	7 (3.0)	-	7 (3.1)	6 (3.3)	-
Fang-Beti	200 (48.7)	75 (43.6)	125 (52.3)	-	99 (43.2)	101 (55.5)	-
Foreigner	8 (1.9)	4 (2.3)	4 (1.7)	-	5 (2.2)	3 (1.7)	-
Marital status, n (%)				0.01^a^			< 0.01^a^
Single	213 (51.8)	99 (57.6)	114 (47.7)	-	160 (69.6)	53 (29.0)	-
Divorced	3 (0.7)	2 (1.2)	1 (0.4)	-	1 (0.4)	2 (1.1)	-
Widowed	17 (4.1)	1 (0.6)	16 (6.7)	-	0	17 (9.8)	-
Married	137 (33.3)	55 (32.0)	82 (34.3)	-	47 (20.9)	90 (49.2)	-
Cohabitation	41 (10.0)	15 (8.7)	26 (10.9)	-	21 (9.1)	20 (10.9)	-
Housing				0.05			0.05^a^
Alone	40 (9.7)	24 (14.0)	16 (6.7)	-	22 (9.6)	18 (9.9)	-
With family	356 (86.6)	142 (82.7)	214 (89.5)	-	194 (84.7)	162(83.0)	-
Shared pit	15 (3.6)	6 (3.5)	9 (3.8)	-	13 (5.7)	2(1.1)	-
Neighbourhood deprivation				0.63			0.62
Low deprivation	174 (42.3)	70 (40.7)	104 (43.5)	-	135 (59.0)	102 (56.0)	-
High deprivation	237 (56.7)	102 (59.3)	135 (56.5)	-	94 (41.0)	80 (44.0)	-

Burden of major CVD risk factors

Distribution of Key NCD Risk Characteristics of the Study Population

The AUDIT-C score for harmful alcohol consumption was significantly higher among male participants (median = 4). Additionally, male participants reported higher levels of commuting for leisure (median = 290 min/week) and having a higher moderate-to-vigorous physical activity (MVPA) level (median = 360 min/week). Furthermore, female participants were found to have higher BMI (median = 26.4 kg/m^2^), waist circumference (median = 94cm), and fat mass percentage (median = 33.4%), while males had higher systolic and diastolic blood pressure (median = 122 mmHg and 78 mmHg) and highest overall cardiovascular risk (median = 9).

With respect to age groups, participants aged 30 years and younger reported a lower AUDIT-C score (median = 1), higher leisure active commuting (median = 270 minutes/week), and MVPA (median = 30 min/week). Additionally, they were found to have lower BMI (median = 23.9 kg/m^2^) and overall cardiovascular risk score (median = 7). Table 3 displays the median value of NCD risk factors by gender and age group.

**Table 3:** Distribution of key NCD risk characteristics of the study population AUDIT-C score: Alcohol Use Disorder Identification Test-Consumption score; IQR: inter-quartile range; MVPA: moderate-to-vigorous physical activity; NL-IHMRS: Non-laboratory INTERHEART Modifiable Risk Score

Characteristics	Total	Gender			Age group		
		Male; median (IQR)	Female; median (IQR)	P-value	Up to 30; median (IQR)	Over-30; median (IQR)	P-value
AUDIT-C score	2 (0-5)	4 (0-7)	1 (0-3)	< 0.01	1 (0-4)	3 (0-7)	< 0.01
Active commuting (minutes/week)	210 (70- 525)	290 (90-630)	180 (60-420)	0.01	270 (80-742)	140 (60-420)	< 0.01
MVPA (minutes/week)	300 (120-620)	360 (138.8-720)	240 (120-427.5)	< 0.01	30 (0- 330)	0 (0 -180)	0.02
Tobacco index (pack years)	3 (1.5-10)	2.5 (1.25-10)	5 (5-5)	0.71	2 (1-2)	5 (3.25-17.5)	0.08
Second-hand smoking (hours/week)	0 (0-1)	0 (0-1)	0 (0-1)	0.63	0 (0-0.5)	0 (0-1)	0.57
Exposure to firewood smoke (hours/week)	0 (0-3)	0 (0-0)	0 (0-5)	< 0.01	0 (0-2)	0 (0-3)	0.84
BMI (kg/m2)	25.3 (22.3-30)	24.1 (21.7-27.8)	26.4 (23.4-31.5)	< 0.01	23.9 (21.7-26.6)	28.4 (24.1-32.5)	< 0.01
Mid-arm circumference (cm)	31 (29-34)	30.5 (29-33.5)	31 (28-34)	0.48	30 (28-32)	33 (30-36)	< 0.01
Waist circumference (cm)	91 (83-101)	88 (82-97)	94 (86-103)	< 0.01	87 (81.2-92)	100 (91- 108)	< 0.01
Hip circumference (cm)	105 (96-115)	98 (92-107)	109 (101-118)	< 0.01	100 (93-106)	113 (104-122)	< 0.01
Waist/hip ratio	0.88 (0.86-0.89)	0.90 (0.89-0.90)	0.86 (0.85-0.87)	< 0.01	0.87 (0.85-0.89)	0.89 (0.87 -0.91)	< 0.01
Fat mass percentage (%)	28 (17.6 -36.3)	16.7 (12.4-24)	33.4 (27.9-39.6)	< 0.01	25.1 (15.9-32.7)	31.4 (21.5-39.5)	< 0.01
Systolic blood pressure (mmHg)	115 (107-128)	122 (113-135)	112 (104-122)	< 0.01	112 (104-120)	124 (112-140)	< 0.01
Diastolic blood pressure (mmHg)	76 (69-84)	78 (71-87)	74 (68-81)	< 0.01	71.5 (67-78.4)	81 (74-91.2)	< 0.01
NL-IHMRS	8 (6-11)	9 (6-12)	8 (5-10.5)	0.01	7 (5 -10)	9 (7-12)	< 0.01

Personal and Family History of Chronic Disease

The prevalence of self-reported hypertension was 8.2% (95% CI 5.9-11.3), and the family history of hypertension and diabetes was 32.9% (95% CI 28.6-37.6%) and 27.4% (95% CI 23.2-31.9%), respectively. The prevalences of self-reported personal or family history were evenly distributed across males and females, while the prevalence of self-reported hypertension was higher (13% (95%CI 8.5-18%)) across the over-30 age group (Table 4).

**Table 4:** Personal and family histories

Characteristics	Total		Gender				Age groups			
			Male		Female		Up to 30		Over 30	
	n (%)	95%CI	n (%)	95%CI	n (%)	95%CI	n (%)	95%CI	n (%)	95%CI
Personal history										
Self-reported hypertension	34 (8.2)	5.9-11.3	14(8.1)	4.9-13.2	20(8.3)	5.4-12.5	10 (4.3)	2.1-7.8	24 (13.1)	8.5-18
Self-reported diabetes	8 (1.96)	1-3.8	4 (2.3)	0.9-6.0	4 (1.7)	0.6-4.3	2 (0.9)	0.1-3.1	6 (3.3)	1.2-7.0
Self-reported gout	9 (2.2)	1.1-4.1	4 (2.3)	0.9-6.0	5 (2.9)	0.9-4.8	2 (0.9)	0.1-3.1	7 (3.8)	1.6-7.7
Self-reported other chronic condition	34 (8.2)	5.9-11.3	12 (7.0)	4.0-11.9	22 (9.1)	6.1-13.4	14 (6.1)	3.4-10.0	20 (10.9)	6.8-16.3
HIV infection (self-reported)	1 (0.24)	0.03-1.7	0 (0.00)	0	1(0.41)	0.0-2.9	0	0.0-1.2	1 (0.5)	0.0-3.0
Family history										
Either or both biological parents had a heart attack	18 (4.4)	2.8-6.8	5(2.9)	1.2-6.8	13 (5.4)	3.2-9.1	11 (4.8)	2.4-8.4	7 (3.8)	1.6-7.7
Either or both biological parents had a stroke	31 (7.5)	5.3-10.5	13 (7.6)	4.4-12.6	18 (7.5)	4.5-11.6	11 (4.8)	2.4-8.4	20 (10.9)	6.8-16.4
Either or both biological parents had died suddenly	35 (8.5)	6.1-11.6	18 (10.5)	6.7-16.0	17 (7.0)	4.4-11.1	17 (7.3)	4.4-11.6	18 (9.8)	5.9-16.1
Either or both biological parents have/had diabetes	113(27.4)	23.2-31.9	41 (23.8)	18.0-30.7	72 (29.9)	24.4-38.0	55 (23.9)	18.6-30.0	58 (31.7)	29.0-35.0
Either or both biological parents have/had hypertension	136 (32.9)	28.6-37.6	52 (30.2)	23.8-37.5	84 (34.9)	29.1-41.1	69 (30.0)	24.2-36.4	67 (36.6)	29.6-44.0
Diastolic blood pressure (mmHg)	76 (69-84)	78 (71-87)	74 (68-81)	< 0.01	71.5 (67-78.4)	81 (74-91.2)	< 0.01			
NL-IHMRS	8 (6-11)	9 (6-12)	8 (5-10.5)	0.01	7 (5 -10)	9 (7-12)	< 0.01			

Alcohol, Tobacco and Mental Health

Table 5 displays the estimated prevalences of alcohol, tobacco smoking and mental health in our study population. The overall estimate for hazardous alcohol use was 40.4% (95% CI 35.8-45.3). Notably, men had a higher consumption of alcohol at hazardous levels than women (55.2% (95% CI 47.7-62.5%) vs. 29.9% (95% CI 24.4-40). On the other hand, participants over 30 years of age were also found to have a higher prevalence of hazardous alcohol consumption (50.8% (95% CI 43.3-58.3) vs 32.2% (95% CI 26.2-38.6)).

**Table 5:** Alcohol, tobacco, and mental health of the population by gender and age group

Characteristics	Total		Gender				Age groups			
			Male		Female		Up to 30		Over 30	
	n (%)	95%CI	n (%)	95%CI	n (%)	95%CI	n (%)	95%CI	n (%)	95%CI
Lifestyle										
Hazardous alcohol use	167 (40.4)	35.8-45.3	95 (55.2)	47.7-62.5	72 (29.9)	24.4-40	74 (32.2)	26.2-38.6	93 (50.8)	43.3-58.3
Tobacco smoking										
Former	19 (4.6)	2.9-7.1	17(9.9)	6.2-15.3	2 (0.8)	0.2-3.3	10 (4.3)	2.1-7.3	14 (7.7)	4.2-12.5
Current	21 (5.1)	3.3-7.7	19(11.1)	7.1-16.7	2 (0.8)	0.2-3.3	9 (3.9)	1.8-7.3	10 (5.5)	2.7-9.8
Second-hand smoking	110 (26.6)	22.6-31.1	47 (27.3)	21.2-34.5	63 (26.1)	21.0-32.1	64 (27.8)	22.1-34.1	46 (25.1)	19.0-32.1
Regular exposure to firewood smoke	130(31.5)	27.2-36.1	26(15.1)	10.5-21.3	104(43.2)	37.0-49.5	73 (31.7)	27.8-38.1	57 (31.1)	24.5-38.4
Mental health										
Experienced anxiety over the last 12 months	345 (83.5)	79.6-86.8	149 (86.6)	80.7-91	196 (81.3)	75.9-85.8	183 (79.6)	73.8-84.6	162 (88.5)	83-92.8
Felt sad, melancholy or depressed for 2 weeks or more in a row	216 (52.3)	47.4-57.1	77 (44.8)	37.5-53.3	139 (57.7)	51.3 -63.8	109 (47.4)	40.8-54.1	107 (58.5)	51.0-65.7

The overall prevalence of current and former smoking was 4.6% (95% CI 2.9-7.1%) and 5.1% (95% CI 3.3-7.7), respectively. Men had a significantly higher prevalence of both current and former smoking status, while females reported a higher prevalence of exposure to firewood smoke than men (43.2% (95% CI 37.0-49.5%) vs. (15.1% CI 10.5-21.3%). We did not find any significant differences between the two age groups.

The overall prevalence of self-reported anxiety and depression was 83.5% (95% CI 79.6-86.8%) and 52.3% (95% CI 47.4-57.1%), respectively. Women and participants over 30 years old of age showed a higher prevalence of depression.

Diet and Physical Activity

The prevalence of low active commuting in our study population was 34.1% (95% CI 28.7-37.8), with a higher proportion of women falling into this category (38.1% (95% CI: 32.2-44.4%) vs. 26.2% (95% CI: 20.2-33.2%)), while men were more likely to be categorised as highly active commuters with a prevalence of 32.6% (95% CI: 26.0-39.9%) vs 22.6% (95% CI: 17.8-28.3%) (Table 6). 

**Table 6:** Diet and physical activity behaviours by gender and age groups Tertile 1: < 120 min/week; Tertile 2: 120 to 420 min/week; Tertile 3: Over 420 min/week MVPA: moderate-to-vigorous physical activity. The recommended level of MVPA is ≥ 150 min/week.

Characteristics	Total		Gender				Age groups			
			Male		Female		Up to 30		Over 30	
	n (%)	95%CI	n (%)	95%CI	n (%)	95%CI	n (%)	95%CI	n (%)	95%CI
Physical activity										
Active commuting per week (min/week)										
Tertile 1 (Lowest commuters)	136 (33.1)	28.7-37.8	47 (26.2)	20.2-33.2	91 (38.1)	32.2–44.4	70 (30.6)	25.0-36.8	66(31.3)	29.6-43.5
Tertile 2 (Mild commuters)	165 (40.1)	35.5-45.0	71 (41.3)	34.2-48.7	94 (36.3)	33.4-45.6	90 (39.3)	33.2-45.8	75 (41.2)	34.3-48.5
Tertile 3 (highest commuters)	110 (26.8)	22.7-31.2	56 (32.6)	26.0-39.9	54 (22.6)	17.8-28.3	69 (30.1)	24.6-36.4	41 (22.5)	17.1-29.1
MVPA per week										
Not recommended level	273(66.4)	61.7-70.8	92 (53.5)	46-60.8	181 (75.7)	69.9-80.7	143 (62.4)	56.0-68.5	130 (71.4)	64.5-77.5
Recommended level	138(33.6)	29.2-38.3	80 (46.5)	39.2-54.0	58 (24.3)	19.3-30.1	86 (37.6)	31.5-44.0	52 (28.6)	22.5-35.5
Diet and nutrition										
Eating salty food or snacks one or more times a day	385(93.2)	90.3-95.3	161 (93.6)	88.8-96.4	224 (93.0)	88.9-95.6	218 (94.8)	91.1-97.3	167 (91.3)	86.2-94.9
Eating foods/drinks containing too much sugar one or more times a day	245(59.3)	54.5-64	168 (62.2)	54.7-69.2	105 (57.3)	50.9-63.4	151 (65.7)	59.1-71.8	94 (51.4)	43.9-58.8
Eating oily foods/snacks one or more times a day	192(46.5)	41.7-51.3	89 (51.7)	44.3-59.1	103 (42.7)	36.6-49.1	117 (50.9)	44.2-57.5	75 (41.0)	33.8-48.5
Eating oily foods/snacks three or more times a week	247(59.8)	55-64.4	116 (67.4)	60.0-74	141 (54.4)	48-60.6	141 (61.3)	54.7-67.6	106 (57.9)	50.4-65.2
Eating dairy products one or more times a day	170(41.1)	36.5-46	72 (41.2)	34.7-49.4	98 (40.7)	34.6-47	108 (47.0)	40.4-53.6	62 (33.9)	27.1–41.2
Eating dairy products three or more times a week	254(61.5)	56.7-66.1	113 (65.7)	58.3-72.4	141 (58.5)	52.2-64.6	154 (67.0)	60.5-73.0	100 (54.6)	47.1-62.0
Eating deep-fried foods or snacks or fast foods three or more times a week	206(49.9)	45.1-54.7	84 (48.8)	41.4-56.3	122 (50.6)	44.3-56.9	134 (58.3)	51.6-64.7	72 (39.3)	32.2-46.8
Eating meat and/or poultry two or more times daily	185(44.7)	40-49.6	83 (49.4)	40.9-55.7	102 (52.7)	36.2-48.7	111 (48.3)	41.6-54.9	74 (40.4)	33.3-47.9
Not eating at least 1–2 cups/portions of fruits one or more times a day (inadequate fruit consumption)	212(51.3)	46.5-56.1	85 (49.4)	42-56.9	127 (52.7)	46.4-58.9	105 (45.7)	39.1-52.3	107 (58.4)	51.0-65.7
Not eating at least 2–3 cups of vegetables one or more times a day (inadequate vegetable consumption)	250(60.5)	55.7-65.1	112 (65.1)	57.7-71.9	138 (57.2)	50.9-64.4	155 (67.4)	60.1-73.4	95 (51.9)	44.4–59.3
Inadequate fruit and vegetable consumption	113(36.6)	31.4-42.1	52 (37.1)	29.5-45.5	61 (36.1)	29.2-43.6	72 (31.3)	25.4-37.7	59 (32.2)	25–37.7

The overall recommended MVPA prevalence in our study population was 33.6% (95% CI 29.2-38.3%). Moreover, Males were more likely to engage in physical activity at a recommended level (150 min/week) with a prevalence of 46.5% (95% CI: 39.2-54.0%) vs 24.3% (95% CI: 19.3-30.1%).

Furthermore, the prevalence of inadequate vegetable consumption was higher among the up to 30-year-old age group 67.4% (95% CI 60.1-73.4%) vs 51.9% (95% CI 44.4-59.3%). However, when we considered both fruit and vegetable consumption, we found that 36.6% (95% CI 31.4-42.1%) of our participants consumed inadequate amounts of fruits and/or vegetables, with no notable disparity between male and female participants and both age groups.

Among our participants, 93.2% (95% CI 90.3-95.3%) reported eating salty food at least once per day, 59.3% (95% CI 54.5-64%) reported eating food and/or drinks containing sugar at least once per day, 46.5% (95% CI 41.7-51.3%) reported eating oily food or snacks more than once per day. Additionally, 41.1% (95% CI 36.5-46%) reported eating dairy food at least once per day, 49.9 % (95% CI 45.1-54.7%) reported eating fried foods or three or more times a week, and 44.7% (95% CI 40-49.6%) reported eating meat and/or poultry at least two times daily.

We did not find any significant differences in diet patterns among male and female participants. However, among the participants in their thirties, we found a higher prevalence of daily consumption of sugary food and drinks (65.7% (95% CI 59.1-71.8) vs 51.4 % (95%CI 43.9-58.8)) and dairy foods (47.0% (95% CI 40.4-53.6) vs 33.9% (95% CI 27.1-41.2)).

Metabolic Syndrome and the Global Cardiovascular Risk

According to our participants' BMI, 27% (95% CI: 22.9 to 31.5) were overweight and 25.1% (95% CI: 21.2 to 29.6%) were obese (Table 7). We found a higher prevalence of obesity among females (33.1% (95% CI 27.4-39.2%) vs. 14% (95%CI 9.5-20%)) and among participants over 30 years of age (41.5 (95% CI 34.3-48.5%) vs. 12.2% (95%CI 8.6-17.1%)). Additionally, the overall prevalence of abdominal obesity (using IDF criteria) and excess body fat mass was 62.2% (95% CI: 57.4 to 66.8%) and 46.3% (95% CI: 41.4-51.1%) respectively. Furthermore, females and participants over 30 years of age were found to have a higher prevalence of abdominal obesity according to both IDF criteria and WHO criteria, and a higher prevalence of excess body fat mass. The overall prevalence of suspected prehypertension and hypertension was 58.3% (95% CI 57.4-66.8) and 41.8% (95% CI 35-48.9), respectively. No significant differences were found between men and women, while participants over 30 years of age had a significantly higher prevalence of both suspected prehypertension and hypertension (Table 7).

**Table 7:** Metabolic syndrome and 10 year-cardiovascular risk by gender and age groups

Characteristics	Total		Gender				Age groups			
			Male		Female		Up to 30		Over 30	
	n (%)	95%CI	n (%)	95%CI	n (%)	95%CI	n (%)	95%CI	n (%)	95%CI
Anthropometric parameters and blood pressure										
Body mass index (kg/m2)										
Underweight (<18.5)	6(1.5)	0.7-3.2	3 (1.7)	0.6-5.3	3(1.3)	0.4-3.6	2 (0.9)	0.2-3.1	4 (2.2)	0.9-5.5
Normal (18.5–24.9)	191(46.5)	41.7-51.3	96(55.8)	48.3-63.1	95(39.7)	33.8-46.1	139 (60.7)	54.2-68.8	52 (28.6)	22.5-35.5
Overweight (25–29.9)	111(27.0)	22.9-31.5	49(28.5)	22.2-35.7	62(25.9)	20.8-31.8	60 (26.2)	20.9-32.3	51 (28.0)	22.0-34.9
Obesity (≥30)	103(25.1)	21.1-29.5	24(14.0)	9.5-20	79(33.1)	27.4-39.2	28 (12.2)	8.6-17.1	75 (41.2)	34.3-48.5
Abdominal obesity and excess body fat mass										
Abdominal obesity using WC (IDF criteria)	272(65.9)	61.1-70.2	55(32.0)	25.4-39.3	217(90.0)	85.5-93.2	127 (55.2)	48.5-61.7	145 (79.2)	72.6-84.9
Abdominal obesity using WHR (≥0.9/0.85 for men/women)	257(62.2)	57.4-66.8	71(41.3)	34.1-48.8	186(77.2)	71.4-82.1	112 (48.7)	42.1-55.4	145 (79.2)	72.6-84.9
Excess body fat mass (>20/33% for men/women)	191(46.3)	41.4-51.1	65(37.8)	30.8-45.2	126(52.3)	46-58.5	70 (30.4)	24.6-36.8	121 (66.1)	58.7- 72.9
Systolic blood pressure										
Normal	251(61.1)	56.3-65.7	78(45.3)	38-52.9	173(72.4)	66.4-77.7	170 (74.2)	68.2-79.9	81 (44.5)	37.5-51.8
Prehypertension	104(25.3)	21.3-29.7	59(34.3)	27.6-41.5	45(18.8)	14.4-24.3	49 (21.4)	16.6-27.2	55 (30.2)	24.0-37.2
Hypertension	56(13.6)	10.6-17.3	35(20.4)	15-27	21(8.8)	5.8-13.1	10 (4.4)	2.4-7.9	46 (25.3)	19.5-32.1
Diastolic blood pressure										
Normal	263(64.0)	59.2-68.5	96(55.8)	48.3-63.0	167(69.9)	63.8-75.3	181 (79.0)	73.3-83.8	82 (45.1)	38.0-52.3
Prehypertension	85(20.7)	17.0-24.9	45(26.2)	20.2-33.2	40(16.7)	12.5-22.0	37 (16.2)	12.0-21.5	48 (26.4)	20.5-33.2
Hypertension	63(15.3)	12.2-19.1	31(18.0)	13-24.4	32(13.4)	9.7-18.3	11 (4.8)	2.7-8.4	52 (28.6)	22.5-35.5
High blood pressure										
Suspected prehypertension (SBP 120-139 or DBP 80-89 mm Hg)	113(58.3)	51.1-65	61(59.2)	49.4-68.3	52(57.1)	46.8-66.9	56 (75.7)	64.3-84	57 (47.5)	38.3-56.8
Suspected hypertension (SBP ≥140 or DBP≥90 mm Hg)	81(41.8)	35-48.9	42(40.8)	31.7-50.6	39(42.9)	33.1-53.2	18 (24.3)	15.1-35.7	63 (52.5)	43.2-61.7
10-year cardiovascular risk										
Low risk	251 (61.1)	56.3-65.7	95 (55.2)	47.8-22.5	156 (65.3)	59.0-71.0	157 (68.6)	62.3-74.2	94 (51.6)	44.4-58.8
Moderate risk	131 (31.9)	27.6-36.5	60 (34.9)	28.2-42.3	71 (29.7)	24.3-35.8	64 (27.9)	22.5-34.1	67 (36.8)	30.1-44.0
High risk	29 (7.1)	5.0-10.0	17 (9.9)	6.3-15.3	12 (5.0)	2.9-8.6	8 (3.5)	1.8-6.7	21 (11.5)	7.7-17.0

We computed the 10-year risk of cardiovascular events, using the NL-IHRMS. Our results showed that 35.6% (95% CI 31.0-40.4%) of our participants had a moderate risk of cardiovascular disease and 8.7% (95% CI 6.2-11.9%) had a high risk. We also noted a non-significant higher prevalence of both moderate and high risks of cardiovascular disease among males. Furthermore, the prevalence of moderate risk and high risk of cardiovascular disease was (27.9% (95% CI 22.5-34.1%) and 3.5% (95% CI 1.8-6.7%), respectively, among up to 30-year-old participants. However, these prevalence rates were significantly lower than those of the participants over 30 years of age.

Assessment of other factors that could independently influence cardiovascular disease risk 

Multicollinearity Assessment

**Figure 2:**
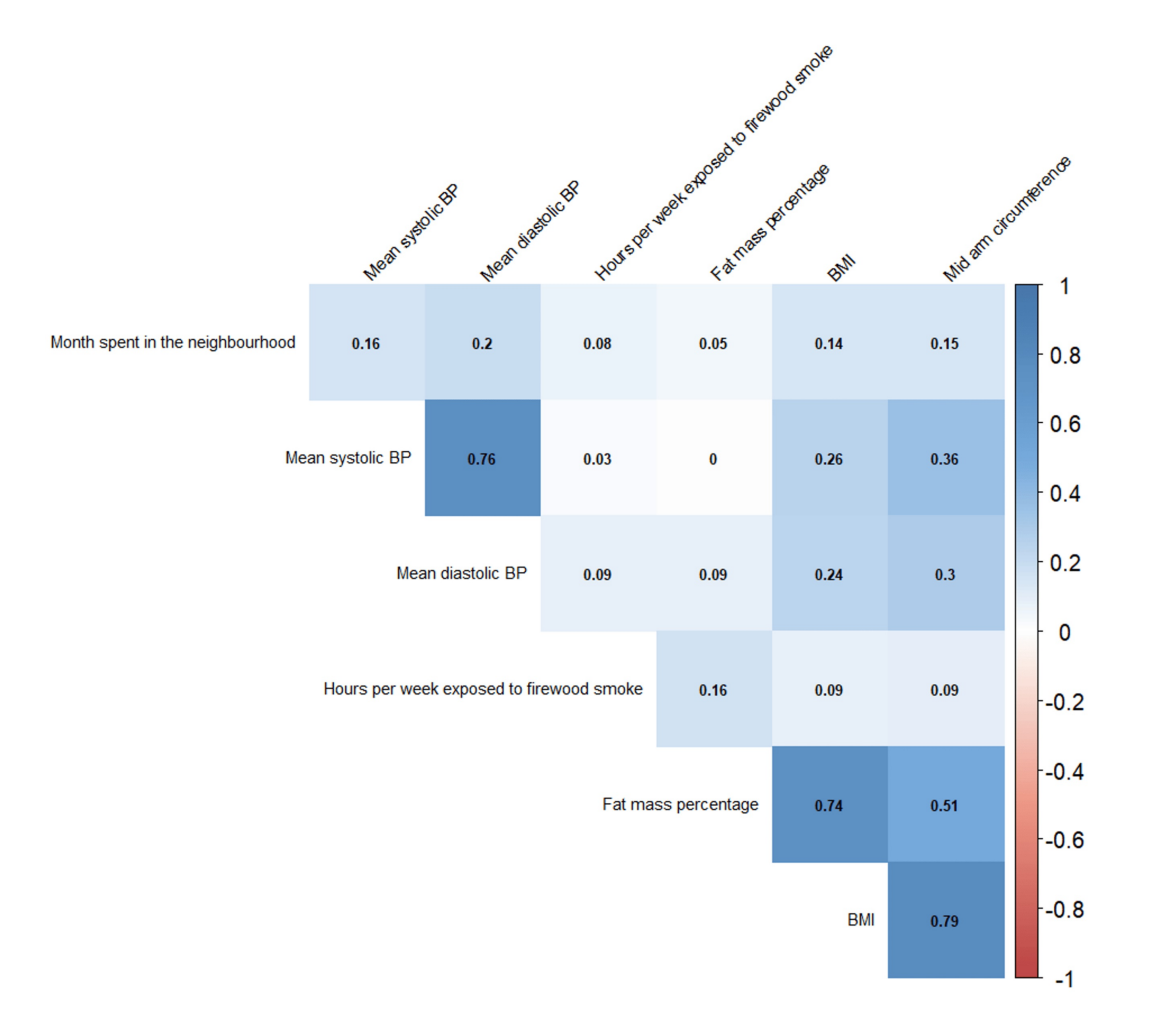
Spearman's correlation plot for continuous variables

The highest significant correlations found between continuous parameters were for BMI and mid-arm circumference (ρ=0.79; p<0.01), followed by systolic and diastolic blood pressure (ρ=0.76; p<0.01), BMI and fat mass percentage (ρ=0.75; p<0.01), and mid-arm circumference and fat mass percentage (ρ=0.51; p<0.01) (Figure 2). NL-IHRMS also showed a poor but significant correlation with various continuous variables that were not used to compute the score.

**Figure 3:**
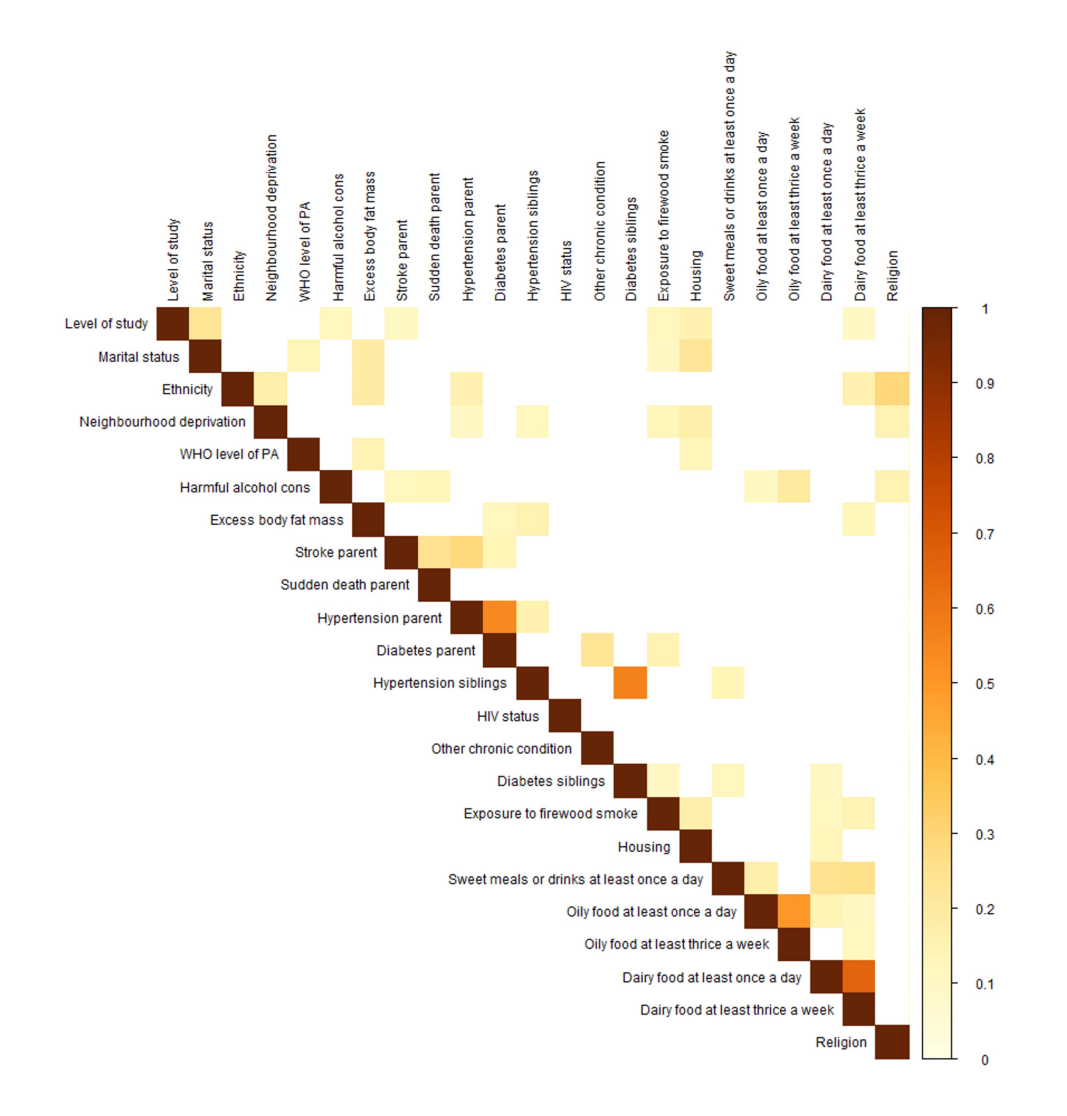
Cramer V plot for categorical variables

We also tested the correlation between categorical parameters and found a high correlation between the self-reported prevalence of hypertension and diabetes among biological parents (V=0.545, p < 0.01), and among siblings (V = 0.560, p < 0.01). We also found a high correlation between those eating dairy foods once a day and at least three times a week (V= 0.660, p < 0.01), and oily foods once a day and at least three times a week (V= 0.504, p < 0.01) (Figure 3).

For the regression analysis, mid-arm circumference was dropped due to less clinical importance, and systolic and diastolic blood pressure were combined to mean arterial pressure. Furthermore, self-reported prevalence of hypertension and diabetes among parents, hypertension and diabetes among siblings and oily food patterns were also combined into a single variable and fat mass percentage was categorised.

Additional Factors Influencing Cardiovascular Risk

Table 8 presents the results of univariable and multivariable regression analyses, examining other factors that might have independently affected CVD risk in the study population. The multivariate model showed that a personal history of diabetes, hypertension, or other chronic diseases, alongside behaviours like binge drinking, can significantly increase the risk of CVD in our study population. Furthermore, mean arterial pressure also influences cardiovascular risk.

**Table 8:** Univariable and multivariable ordinal logistic regression analyses of independent factors that may have influenced the cardiovascular disease risk Significance * p < 0.05 Adjusted for BMI, mean blood pressure and significant variables from the univariate model AUDIT-C score: Alcohol Use Disorder Identification Test-Consumption score; MVPA: moderate-to-vigorous physical activity

Variables	Odds ratio	95% CI	Adjusted odds ratio	95% CI
BMI	1.028	0.974-1.086	1.036	0.982-1.092
Mean arterial pressure	1.018	1.002-1.034*	1.017	1.001-1.032*
Audit C Score: no harmful alcohol consumption	0.496	0.308-0.793*	0.486	0.307-0.767*
MVPA: not recommended level	1.189	0.742-1.920	-	-
Level of study: Undergraduate	1.293	0.978-2.174	-	-
No excess body fat mass	1.112	0.617-2.021	-	-
Marital status: In a couple	1.363	0.859-1.163	-	-
Religion (Ref: Christian)				
Muslim	1.113	0.428-2.769	-	-
Other	1.590	0.480-4.960	-	-
Ethnicity (Ref: Bantu)				
Grass Field	1.122	0.689-1.826	-	-
Sawa	1.037	0.254-3.803	-	-
Sudano-Sahelian	0.858	0.368– 1.920	-	-
Foreigner	1.368	0.019-2.307	-	-
Neighbourhood deprivation: High	0.757	0.478-1.191	-	-
Duration in the neighbourhood	1.001	0.999-1.003	-	-
Housing (Ref: Alone)				
With family	0.980	0.452-2.181	-	-
Shared pit	1.693	0.469-5.975	-	-
Not exposed to firewood smoke	1.305	0.708-2.434	-	-
Exposure to firewood smoke (hrs/week)	1.001	0.962-1.040	-	-
No history of other chronic disease	0.412	0.197-0.864*	0.433	0.214-0.884*
No history of stroke among biological parents	0.633	0.285-1.420	-	-
No history of sudden death among biological parents	0.623	0.414- 1.556	-	-
No history of either high blood pressure or diabetes among biological parents	0.562	0.351-0.898*	0.572	0.563-0.903*
No history of either high blood pressure or diabetes among siblings	0.630	0.357-1.117	-	-
Not eating sweet meals/food/drinks at least once a day?	0.898	0.568-1.413	-	-
Not eating oily foods or snacks once a day or at least 3 times a week	0.797	0.496-1.270	-	-
Not eating dairy foods once a day or at least 3 times a week	1.134	0.714-1.796	-	-

## Discussion

This study measured the significant presence of key CVD risk factors and assessed the global cardiovascular risk and the factors that may influence it in the studied population in Yaoundé.

Tobacco smoking

The prevalence of current smokers was nearly equal to nationwide surveys in the same age group, such as the Global Adult Tobacco Survey in 2013 and the STEPS Survey in 2003, in which the prevalence of current tobacco smokers was 6% and 6.3%, respectively. Male participants had a higher tendency to be smokers, as reported in sub-Saharan countries [23]. Evidence shows that males have higher smoking behaviours, smoke more cigarettes daily, and are less likely to intend to quit smoking compared to females, who are more concerned about the harm to health caused by smoking and have a higher intention to quit smoking [24]. This trend seems to reverse with age; in older age groups, the prevalence of tobacco use seems to be higher among women [25]. 

In addition, the proportion of people reporting exposure to secondhand smoke is lower than that in the Global Adult Tobacco Survey (GATS) for the Cameroon territory, which reports a higher proportion of exposure to secondhand smoke. This finding may seem surprising given that Cameroon ratified the Framework Convention of Tobacco Control (FCTC), which includes measures to prevent exposure to secondhand smoke [26]. However, the duration of exposure in our study appears to be quite low compared to the GATS study, which reported high exposure at home, at work, in public places, and on transport. The relatively low duration of exposure in Yaoundé may indicate that implementation of the law might be more effective in Yaoundé. 

Alcohol consumption

Few studies have examined hazardous alcohol consumption in Cameroon, although one study of adults in Yaoundé in 2007 reported a prevalence of about 65.5% among adults in Yaoundé [27]. However, this study focused on only one district in Yaoundé and did not focus on binge drinking but on total alcohol consumption, which may explain this higher prevalence. Nansseu et al. in Yaoundé found that male sex was a significant predictor of hazardous alcohol use [28], albeit with an overall lower prevalence among university students in Yaoundé. However, the findings of Mbatia et al., showed that the peak of hazardous alcohol consumption in sub-Saharan Africa was between 25 and 35 years of age [29]. 

Diet and physical activity

In Cameroon, the consumption of fruits and vegetables is a subject of considerable interest owing to its implications for public health and nutrition. The country is one of the leading producers in French-speaking Africa, with an estimated availability of 158 grams per capita per year, according to Ganry et al [30]. This abundance is reflected in the dietary habits of the Yaoundé population, where over half of households consume vegetables at least once a week, as reported by Kamga [31]. However, the regularity of this consumption varies among adults, from an average intake of fruits on three days and vegetables on 2.4 days per week [27], with daily consumption of approximately 502 grams per day among army defence forces [32].

Moreover, a study of physical activity patterns revealed that the average self-reported duration of moderate to vigorous physical activity (MVPA) per week aligns with findings by Assah et al., who objectively measured leisure physical activity among urban adults and found an average of around 60 minutes per day [33]. Despite the tendency for self-reported physical activity levels to be higher than those measured objectively, the convergence between self-reported and objectively measured activity levels is noteworthy. On the other hand, Nkondjock and colleagues evaluated leisure physical activity in a population of members of defence forces and found an average leisure physical activity of over 9 MET (metabolic equivalent) h/week [32], as recommended by current guidelines [34]. The insights from these studies provide a nuanced understanding of the dietary and physical activity patterns in Cameroon, which is crucial for developing targeted health interventions and policies.

Metabolic syndrome

The prevalence of overweight and obesity among adults in Cameroon is a growing concern, with various studies highlighting the high prevalence of these conditions in both men and women [15]. Addressing the factors contributing to overweight and obesity, such as lifestyle habits and socioeconomic factors, is crucial for developing effective public health interventions to address this issue in Cameroon.

Furthermore, a previous study conducted a decade ago revealed a lower hypertension prevalence in Yaoundé [27], highlighting the increasing prevalence of hypertension in Cameroon, which had risen by two to five times between 1994 and 2003 [35].

Mental health

Few studies have addressed mental health issues in the Yaoundé population amidst a pandemic or with specific health conditions. However, studies conducted in Cameroon underscore a significant burden of anxiety ranging from 40.3 to 49.2% in the adult population [28,32]. These findings emphasize the importance of addressing mental health issues and implementing appropriate support systems to mitigate the impact of stress and depression in Cameroon.

Global cardiovascular risk

Our study revealed that nearly half (45%) of the study participants had a moderate-to-high risk of developing CVD within the next 10 years. More alarmingly, almost a third (31%) of this population was aged 30 years or less, indicating a high prevalence of CVD risk factors among young adults who could progress to a higher risk category if no preventive measures are taken. This finding is consistent with the growing evidence of the rising burden of CVD and its risk factors among young adults in Yaoundé [10,28] and in the Cameroon urban population, and highlights the need for early screening and intervention programs to reduce CVD risk and improve the cardiovascular health of the adult population in Yaoundé.

Strengths and limitations

The findings of this study should be considered considering its limitations. Self-reported declarations may have led to potential information bias, resulting in the underestimation or overestimation of certain estimates. Additionally, the risk factors that necessitated laboratory investigations were not evaluated. Furthermore, the smoking status classification in current and former smokers could lead to misclassification of occasional or recreational smokers. Finally, the nature of the study and the sampling techniques may raise questions regarding the generalisability of the results to the entire adult population of Yaoundé.

Yaoundé is the second most urbanised city in Cameroon and boasts a population with diverse backgrounds and regional origins. Notably, the research team did not implement any restrictions or distinctions when recruiting participants, thereby leading to the assumption that those who did not participate may have shared similar characteristics with those who did. Furthermore, the study used stringent methodological and statistical approaches to answer the research questions.

## Conclusions

This study revealed a high prevalence of major cardiovascular risk factors and significant global cardiovascular disease risk among adults in Yaoundé, Cameroon. It also showed that younger age groups, likely to adopt unhealthy lifestyles, face an increasing burden of NCDs in this rapidly urbanising city. These findings highlight the need for targeted interventions to address the burden of NCDs in Yaoundé, particularly among younger populations. Further studies are needed to estimate the overall cardiovascular risk in sub-Saharan African populations and evaluate the effectiveness and cost-effectiveness of these interventions.

## Appendices

Appendix 1

Table 9 shows the Non-Laboratory-Based INTERHEART Risk score.

**Table 9:** Non-Laboratory-Based INTERHEART Risk score The tool was adapted from the study by McGorrian et al. [9]. * A non-standard definition of family history was used, as this definition was found to be associated with the best model fit in the final non-laboratory-based model

Risk factor	Question		Points for the answer
Age	Are you a man 55 years or older OR woman 65 years or older?		2
	OR Are you a man younger than 55 years or woman younger than 65 years?		0
Smoking: pick the description which matches you best:	I never smoked		0
	OR I am a former smoker (last smoked more than 12 months ago)		2
	OR I am a current smoker, or I smoked regularly in the last 12 months, and I smoke…	1-5 cigarettes per day	2
		6-10 cigarettes per day	4
		11-15 cigarettes per day	6
		16-20 cigarettes per day	7
		More than 20 cigarettes per day	11
Second hand smoke	Over the past 12 months, what has been your typical exposure to other people’s tobacco smoke?	Less than 1 hour or exposure per week or no exposure	0
		OR One or more hours of second-hand smoke exposure per week	2
Diabetes	Do you have diabetes mellitus?	Yes	6
		No or unsure	0
High blood pressure	Do you have high blood pressure?	Yes	5
		No or unsure	0
Family history	Have either or both of your biological parents had a heart attack?*	Yes	4
		No or unsure	0
Waist to hip ratio	Pick one only:	Quartile 1: Less than 0.873	0
		Quartile 2 &3: 0.873-0.963	2
		Quartile 4: greater than or =0.964	4
Psychosocial factors	How often have you felt work or home life stress in the last year? Pick one only	Never or some periods	0
		OR Several periods of stress or permanent stress	3
	During the past 12 months, was there ever a time when you felt sad, blue, or depressed for two weeks or more in a row?	Yes	3
		No	0
Dietary factors: pick one answer for each food group mentioned	Do you eat salty food or snacks one or more times a day	Yes	1
		No	0
	Do you eat deep fried foods or snacks or fast foods 3 or more times a week?	Yes	1
		No	0
	Do you eat fruit one or more times daily?	Yes	0
		No	1
	Do you eat vegetables one or more times daily?	Yes	0
		No	1
	Do you eat meat and/ or	Yes	2
	poultry 2 or more times daily?	No	0
Physical activity	How active are you during your leisure time?	I am mainly sedentary or perform mild exercise (requiring minimal effort)	2
		OR I perform moderate or strenuous physical activity in my leisure time	0
